# Adverse Effects of Glyphosate and Microcystin-LR on Fish Health: Evidence from Structural and Functional Impairments in Zebrafish Gills

**DOI:** 10.3390/ani15162355

**Published:** 2025-08-11

**Authors:** Yidan Zhang, Han Hu, Linmei Song, Zhihui Liu, Junguo Ma, Xiaoyu Li

**Affiliations:** 1 Pingyuan Laboratory, State Key Laboratory of Antiviral Drugs, College of Life Science, Henan Normal University, Xinxiang 453007, China; zyd123456202202@163.com (Y.Z.); huhanhh666@163.com (H.H.); 18238796595@163.com (L.S.); liuzh2958@163.com (Z.L.); 2 Henan International Joint Laboratory of Aquatic Toxicology and Health Protection, Henan Normal University, Xinxiang 453007, China; 041035@htu.edu.cn

**Keywords:** gill damage, glyphosate, mechanism, microcystin-LR, zebrafish

## Abstract

Glyphosate (GLY) and microcystin-LR (MC-LR) frequently co-occur in natural water bodies. In this study, a subacute exposure test was conducted on zebrafish treated with 3.5 mg/L GLY and 35 μg/L MC-LR, individually and in combination, for 21 d to determine their effects on the gills of zebrafish and their potential mechanisms. The hematoxylin and eosin staining and scanning electron microscopy examination results showed that GLY and MC-LR exposure caused structural damage to gills. Biochemical analysis revealed Na^+^-K^+^-ATPase activity decreased, and the levels of reactive oxygen species, 8-hydroxy-2′-deoxyguanosine, and malondialdehyde increased, inducing oxidative damage to DNA and lipids of gills. Meanwhile, the inflammatory and immune function of the gill was significantly influenced, as evidenced by the alteration of the expression of tumor necrosis factor-α, interleukin-1β, complement 3, and immunoglobulin M. RNA-seq results revealed that GLY and/or MC-LR treatment induced transcriptional changes in the fish gills, which may affect various biological functions, and the lipid metabolism disruption potentially involved in the aforementioned process. Integrating histopathological, biochemical, and transcriptomic analyses, this study revealed that both individual and combined exposures to GLY and MC-LR had adverse effects on zebrafish gills, with combined exposure appears to result in more pronounced adverse effects, potentially compromising fish health.

## Simple Summary

The coexistence of two environmental pollutants, glyphosate (GLY, a widely used herbicide) and microcystin-LR (MC-LR, a toxic byproduct from harmful algal blooms) has become an issue that cannot be ignored. The adverse effects of both individual and combined exposures to GLY and MC-LR on aquatic organisms warrant increasing attention. This study demonstrates that exposure to GLY and MC-LR, either individually or in combination, can induce toxic effects on fish gill structure, potentially impairing their physiological function and ultimately threatening fish survival. Overall, the findings of this study not only enhance our understanding of the detrimental impacts of these pollutants on aquatic animals but also provide valuable insights for ecological and health risk assessments.

## 1. Introduction

Glyphosate (GLY) is an effective broad-spectrum herbicide that is widely used in agricultural systems worldwide [1]. Its mechanism of action involves inhibiting the 5-enol-pyruvylshikimate-3-phosphate synthase enzyme, thereby blocking the shikimic acid pathway, which is essential in the synthesis of aromatic amino acids in fungi, some microorganisms, and plants [2]. Despite the absence of the target enzyme in animals, growing concerns have emerged about the potential adverse effects of residual GLY on animal and even human health, as its widespread use and reliance continue to increase annually [3,4]. The aquatic ecosystem is a crucial reservoir for GLY residues [5], and its widespread application, combined with high water solubility and long environmental half-life, contributes to elevated toxicological risks for aquatic organisms [6,7]. Previous studies have shown that long-term exposure to GLY leads to redox imbalance and metabolic dysfunction in the liver of tilapia and juvenile common carp [8,9]. GLY exposure can also cause developmental toxicity in zebrafish larvae, which might be attributed to abnormal expression patterns of hypothalamic–pituitary–thyroid and growth hormone/insulin-like growth factor axis-related genes, endoplasmic reticulum stress, oxidative stress, inflammation, and apoptosis [10]. Adult zebrafish exposed to GLY exhibit significant impairments in exploration and social behaviors [11]. Therefore, the negative impact of GLY on aquatic animals and aquatic environments should be closely monitored.

In recent years, the global occurrence and severity of cyanobacterial blooms have increased due to eutrophication, raising widespread concern [12]. The potential link between GLY and eutrophication has attracted the attention of numerous researchers [13,14]. Residual GLY in the environment may also serve as a significant phosphorus source in natural water bodies, thereby contributing to water eutrophication and the proliferation of cyanobacteria [15,16]. The species of *Microcystis* is frequently observed as the dominant harmful algal species within phytoplankton during cyanobacterial blooms, and microcystins (MCs), as secondary metabolites produced by *Microcystis*, are the most harmful cyclic peptide cyanotoxins [17]. Furthermore, the presence of GLY also induces the release of MCs by *Microcystis* [18]. There are more than 300 variants of MCs, with microcystin-LR (MC-LR) being one of the most prevalent and highly toxic forms, frequently observed in cyanobacterial blooms [19,20]. Therefore, in the aquatic environment, GLY may coexist with MC-LR, which causes aquatic animals to be at risk of simultaneous exposure to both GLY and MC-LR. A 2013 study revealed that exposure of the *Unio pictorum* Mussel to GLY and MC-LR in combination could disrupt the expression of proteins associated with oxidative pathways, detoxification processes, and energy metabolism [21]. Co-exposure to GLY and MC-LR significantly altered the intestinal microbiota and miRNA expression profiles of zebrafish [22], and induced neurotoxicity in zebrafish fathers and their offspring [23]. Therefore, the combined exposure of aquatic organisms to GLY and MC-LR warrants further attention and investigation. However, the combined exposure of GLY and MC-LR is rarely documented; therefore, it is imperative to investigate their potential joint effects on various organs of aquatic organisms in order to accurately reflect real environmental conditions.

Fish are crucial organisms that occupy the apex of aquatic food webs and serve as significant indicators of overall ecosystem health in aquatic environments. As an intricate and pivotal multifunctional organ, the gill plays a central role in facilitating gas exchange, maintaining acid–base balance, regulating osmotic homeostasis, and excreting nitrogenous waste in fish [24]. Due to its continuous exposure to the surrounding water and its large surface area where blood in the capillaries interfaces with the external environment, the gill serves as a primary site for the efficient uptake of waterborne pollutants into the fish body [25]. Consequently, the fish gill is commonly regarded as a “valve” for aquatic pollutants entering the fish and is typically the first organ to encounter chemicals dissolved in water [26]. The physiological and structural changes induced by these pollutants are commonly observed in this organ, leading to a range of adverse effects on fish, including respiratory dysfunction, circulatory impairments, immune suppression, reduced growth, and even mass mortality events [27]. Therefore, fish gills have been widely recognized as a key biomonitoring tool for assessing environmental changes and are extensively used in studies evaluating the impact of water pollution on aquatic ecosystems and aquatic organisms [28].

Zebrafish (Danio rerio) is a crucial model organism in aquatic ecotoxicology, providing a well-established and widely accepted system for investigating the toxicological effects and mechanisms of aquatic pollutants [29]. Therefore, in this study, zebrafish gills were selected as a model tissue to investigate the adverse effects and underlying mechanisms of GLY and MC-LR following both individual and combined exposure. The results are expected to enhance our understanding of the detrimental impacts of GLY and MC-LR on fish, highlight the necessity of evaluating combined toxicity of environmental risk assessments, and provide a more comprehensive foundation for assessing the risks posed by these contaminants to aquatic organisms.

## 2. Materials and Methods

### 2.1. Chemicals

The GLY compound (CAS: 1071-83-6), with a purity of 99.5%, was purchased from Aladdin (Shanghai, China). It was dissolved in ultra-pure water and stored at −20 °C until use. MC-LR (CAS: 101043-37-2), with a purity of ≥95%, was bought from Express Technology Co., Ltd. (Beijing, China). A stock solution of MC-LR (1 mM) was prepared using ultra-pure water and stored at −20 °C until further use. All additional reagents employed in this study were of analytical grade.

### 2.2. Zebrafish Maintenance

Adult zebrafish (AB strain, wild type, body length 3.9–4.1 cm) utilized in this study were obtained from the China Zebrafish Resource Center (Wuhan, China) and maintained in our laboratory in a recirculating aquaculture system (14:10 light–dark cycle, 28 ± 1 °C). The fish were fed freshly hatched *Artemia salina* two times a day. All animal procedures were conducted following the protocols approved by the Ethics Committee of Henan Normal University (HNSD-2023-2506).

### 2.3. Experimental Design

Healthy male zebrafish (6 months old) were selected and randomly assigned to four exposure groups (75 fish per group): the culture water (abbreviated as CK), 3.5 mg/L GLY group (GLY), 35 μg/L MC-LR group (MC-LR), and 3.5 mg/L GLY + 35 μg/L MC-LR group (MIX). The concentrations of GLY and MC-LR were selected at environmentally relevant concentrations based on previous research [22]. Each treatment was replicated three times. The fish were randomly reared in glass tanks (40 × 25 × 25 cm) with an effective volume of 25 L and maintained at 28 ± 1 °C under 14:10 light–dark cycle for 21 d following OECD guidelines (OECD 2009) [30], and were fed freshly hatched *A. salina* twice a day, with 10 mL provided to each group per meal. During the treatment period, two-thirds of the solution was exchanged every three days, with GLY or MC-LR replenished to the above concentrations. None of the fish died during the exposure period.

Nine zebrafish were randomly selected from each group following 7 and 21 d treatment, and the fish were rinsed with ultrapure water and quickly anesthetized on ice prior to gill dissection using sterile instruments. The gills were stored at −80 °C for subsequent biochemical analysis. Following 21 d of treatment, six additional fish from each group were selected; their gills were prepared for RNA extraction and RNA-seq analysis. Furthermore, gill samples from five other fish per group were collected for histological and scanning electron microscopy (SEM) examination.

### 2.4. Histological Examination

The experimental procedures followed previous research [31]. Fish gills from each group were fixed in 4% paraformaldehyde (PFA) for an overnight period. Following fixation, the samples were dehydrated through a gradient of ethanol (70%, 80%, 90%, and 100%) and were cleared in xylene for transparency. The gill samples were then embedded in paraffin, sectioned at a thickness of 5 μm, stained with hematoxylin and eosin (H&E), and examined under an optical microscopy (Olympus, CX33, Tokyo, Japan).

### 2.5. SEM Examination

The gills of zebrafish were carefully dissected and fixed in 2.5% glutaraldehyde for 24 h at 4 °C. Subsequently, the gills were sectioned to expose the branchial filament, dehydrated through a gradient ethanol series, and immersed in tert-butyl alcohol overnight. The samples were then sputter-coated with gold and examined under a SEM.

### 2.6. Biochemical Analysis

Gill samples were homogenized in ice-cold 0.9% saline at a ratio of 1:9 (*w*/*v*) and subsequently centrifuged at 3000 rpm for 10 min at 4 °C, and the resulting supernatant was collected for biochemical detection [10]. Na^+^-K^+^-ATPase activity and malondialdehyde (MDA) content were determined using biochemical kits (#A070-2-2 and #A003-1-2, respectively; Nanjing Jiancheng, Nanjing, China). Levels of reactive oxygen species (ROS), 8-hydroxy-2′-deoxyguanosine (8-OHdG), tumor necrosis factor-α (TNF-α), interleukin-1β (IL-1β), complement 3 (C3), and immunoglobulin M (IgM) were determined using ELISA kits (#E-335577, #E-42691, #E-43904, #E-43902, #E-42619, and #E-32005, respectively; Andygene, Beijing, China), following the kit’s instructions.

### 2.7. RNA-Seq

The construction and sequencing of RNA-seq libraries from fish gills were performed by Personalbio (Nianjing, China), and the bioinformatics analysis as detailed in Supplementary Methods S1.

### 2.8. Quantitative PCR (qPCR)

RNA extraction from zebrafish gills using the TRNpure Reagent Plus (#NR201-100, Bioconnet, Beijing, China), HiFiScript cDNA Synthesis Kit (#CW2569M, Cwbio Bio., Beijing, China) was then used to reverse-transcribe the RNA into cDNA, and qPCR was performed with Quantagene q225 (Kubo Tech Co., Ltd., Shanghai, China) using 2 × SYBR Premix WizTaq II kit (#NQ811, Nobelab Biotech. Co., Ltd., Beijing, China). The primers utilized in this study are listed in Table S1. Supplementary Methods S2 offers a detailed description of these procedures.

### 2.9. Statistical Analysis

All the statistical analyses were conducted using SPSS 23.0 (IBM, Chicago, IL, USA). The Shapiro–Wilk test and Levene’s test were employed to assess normality and homogeneity of variance, respectively. One-way analysis of variance (ANOVA) was utilized to compare differences among groups.

## 3. Results

### 3.1. Histology of the Gills

**Figure 1:**
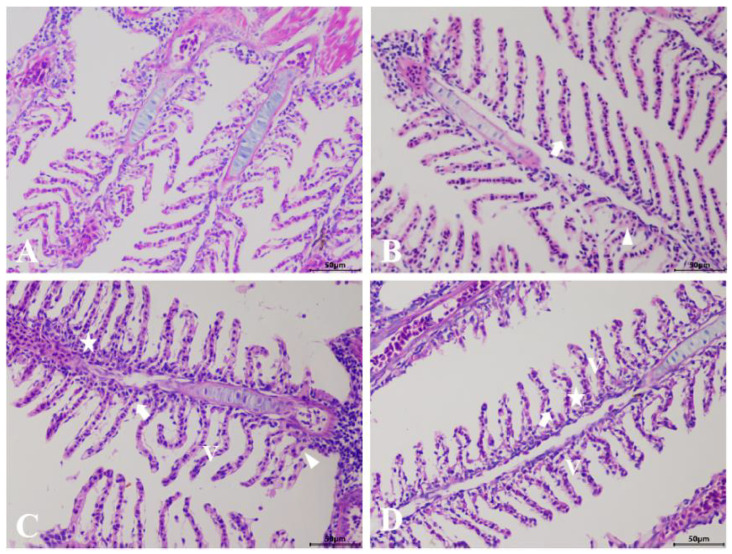
Histological analysis of the gills in zebrafish following GLY and MC-LR exposure. (**A**) Representative images of H&E-stained gills from control groups; (**B**) GLY groups; (**C**) MC-LR groups; (**D**) MIX groups; (V: vacuolation; star: epithelial hyperplasia; triangle: edema; arrow: membrane damage) (Scale bar: 50 μm).

The results of the histological examination revealed that the control group exhibited an intact structure, uniform staining, and regularly arranged lamellar structures in the gills (Figure 1A). Following exposure to GLY and MC-LR, either individually or in combination, H&E staining showed that the gill lamellae of the exposed groups exhibited epithelial hyperplasia and edema, vacuolation, and membrane damage (Figure 1B–D), although the subtle differences in pathological injury among the various exposed groups was insufficient to discern.

### 3.2. SEM of the Gills

**Figure 2:**
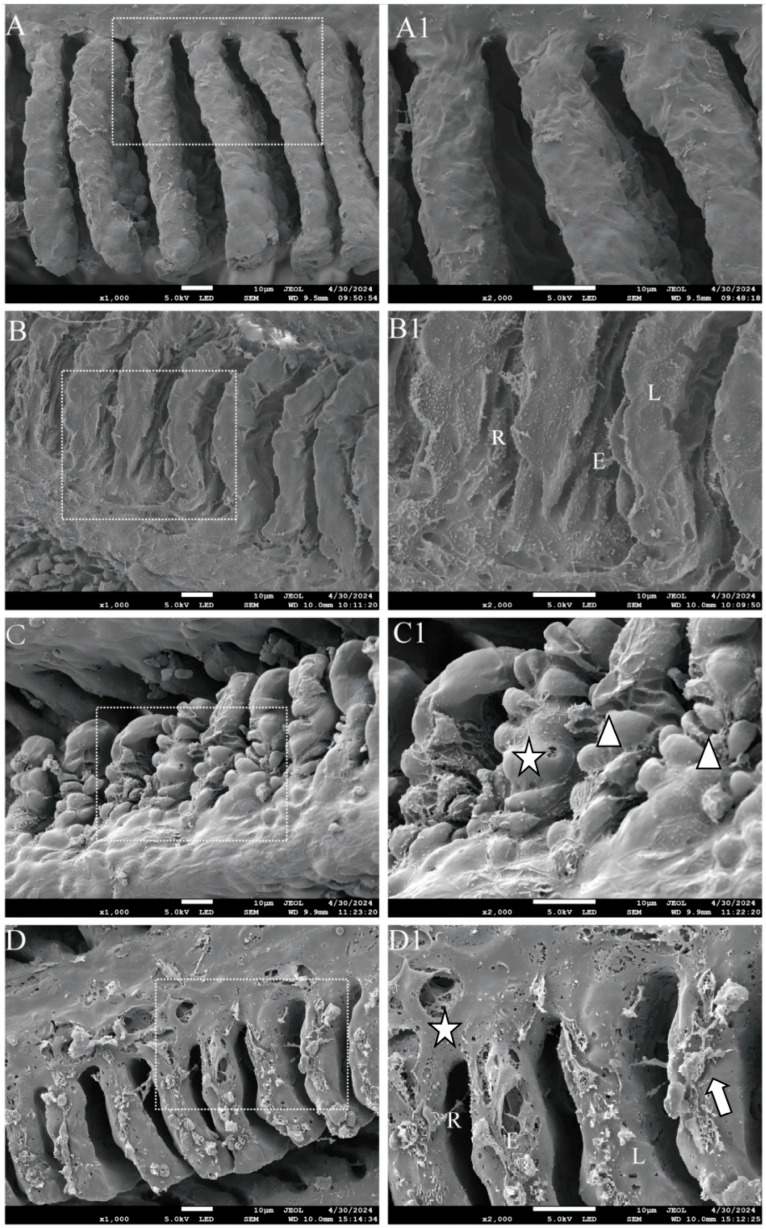
SEM analysis of the gills in zebrafish following GLY and MC-LR exposure. (**A**,**A1**) Representative images of SEM gills from CK groups; (**B**,**B1**) GLY groups; (**C**,**C1**) MC-LR groups; (**D**,**D1**) MIX groups. Scale bar: 10 μm. The image in the second column presents an enlarged version of the white box shown in the first column. Triangle: epithelial hyperplasia of branchial lamella/structural variations; L: lamellae collapse; star: perforation; E: exfoliation of lamellar epithelium; R: reduction of interfilamentary space; arrow: mucous accumulation.

SEM results revealed that the gill filaments in the control group exhibited a normal histological structure, characterized by intact primary and secondary lamellae, with the secondary lamellae being evenly spaced (Figure 2A). GLY exposure caused gill injury in zebrafish, manifested by epithelial exfoliation of gill lamellae and lamellar collapse, resulting in narrowed interlamellar spaces (Figure 2B). Following MC-LR exposure, epithelial hyperplasia or lifting of the gill plate and structural variations were observed in gill filaments, including curling, fusion, and spherically convex morphology of secondary lamellae (Figure 2C). After co-exposure to GLY and MC-LR, the primary gill lamellae exhibited perforations, the gill filament showed distinct sloughing or lifting of the gill epithelium, and the interfilamentary space was reduced, with the surface covered with mucus (mucous accumulation) (Figure 2D).

### 3.3. Na^+^-K^+^-ATPase Activity

**Figure 3:**
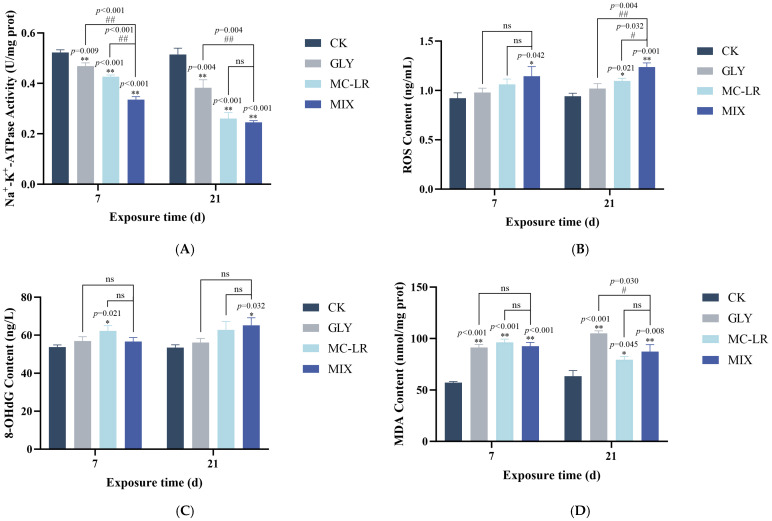
Effects of GLY and MC-LR on the Na^+^-K^+^-ATPase activities and oxidative stress indices of the zebrafish gills. Values are presented as the means ± SD (*n* = 3). Asterisks denote significant differences compared to the controls (* *p* < 0.05, ** *p* < 0.01), and hash symbol represents significant differences between single and combined exposure groups (# *p* < 0.05, ## *p* < 0.01, ns: no significant). (**A**) The activities of Na^+^-K^+^-ATPase in the fish gills; (**B**) the levels of ROS; (**C**) 8-OHdG content; (**D**) MDA content.

The Na^+^-K^+^-ATPase activity in the zebrafish gills showed a significant decrease in all treatment groups at both 7 and 21 d, and the inhibitory effect of MIX exposure on Na^+^-K^+^-ATPase activity was stronger than that observed with GLY or MC-LR single exposure (Figure 3A).

### 3.4. Oxidative Stress-Related Index

The levels of ROS in the GLY-treated groups exhibited an upward trend, albeit not significantly, compared to the controls (Figure 3B). In the MC-LR-treated groups, ROS levels showed an upward trend but were not significant at 7 d, while significantly increased at 21 d (Figure 3B). In the MIX groups, ROS levels were significantly elevated at both 7 and 21 d (Figure 3B). Notably, ROS levels in the gills were generally lower following single treatment to GLY or MC-LR compared to the MIX groups (Figure 3B). Meanwhile, the 8-OHdG content in the zebrafish gills was generally elevated in all treatment groups on both 7 and 21 d. However, this increase was statistically significant only in the MC-LR exposure groups at 7 d and in the combined exposure groups at 21 d (Figure 3C). Furthermore, the MDA contents in the gills of GLY and MC-LR single or co-exposure fish were significantly enhanced at both 7 and 21 d (Figure 3C), and while the MDA content did not show significant alterations between the GLY or MC-LR treated groups and the MIX treated groups, there was a higher level in the GLY group compared to the MIX group at 21 d (Figure 3D).

### 3.5. Inflammation-Related Index

**Figure 4:**
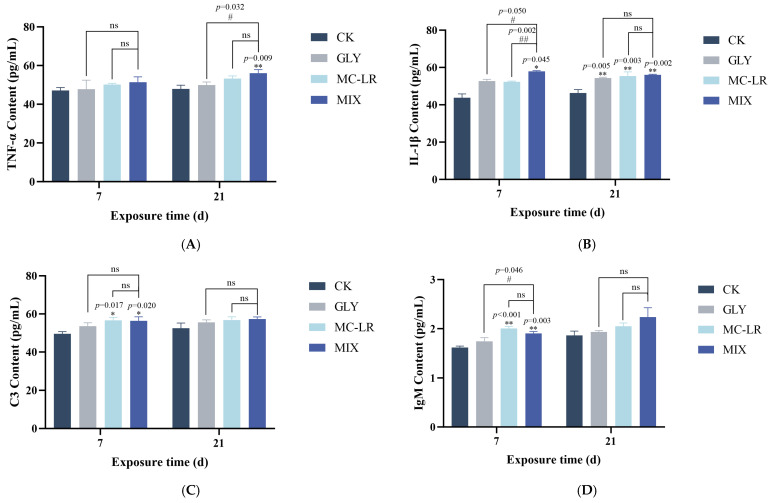
Effects of GLY and MC-LR on the inflammatory and immune related indices of the zebrafish gills. Values are presented as the means ± SD (*n* = 3). Asterisks denote significant differences compared to the controls (* *p* < 0.05, ** *p* < 0.01), and hash symbol represents significant differences between single and combined exposure groups (# *p* < 0.05, ## *p* < 0.01, ns: no significant). (**A**) The levels of TNF-α in the fish gills; (**B**) the levels of IL-1β; (**C**) C3; (**D**) IgM.

As shown in Figure 4A, the TNF-α content in the gills was generally elevated in all three treatment groups compared to the control group; however, this increase was statistically significant only in the combined exposure groups at 21 d. Following a 7 d exposure to GLY and MC-LR, either individually or in combination, IL-1β levels in the gills were generally upregulated; however, this increase was statistically significant only in the combined exposure group (Figure 4B). After 21 d of exposure, IL-1β content was significantly increased in all three treatment groups compared to the control group, with no significant difference in IL-1β levels between the single exposure groups and the combined exposure group (Figure 4B).

### 3.6. Immune-Related Index

The C3 contents in the gills generally increased across all treatment groups; however, statistically significant increases were observed only in the MC-LR and combined exposure groups at 7 d (Figure 4C). Compared to the control group, IgM levels showed no significant change in the GLY-treated groups at either 7 or 21 d (Figure 4D). Following MC-LR exposure, IgM content was significantly upregulated at 7 d, but no significant alteration was observed at 21 d (Figure 4D). In the MIX groups, the variational trend of IgM was similar to that observed in the MC-LR-treated groups (Figure 4D).

### 3.7. Transcriptome Analysis

**Figure 5:**
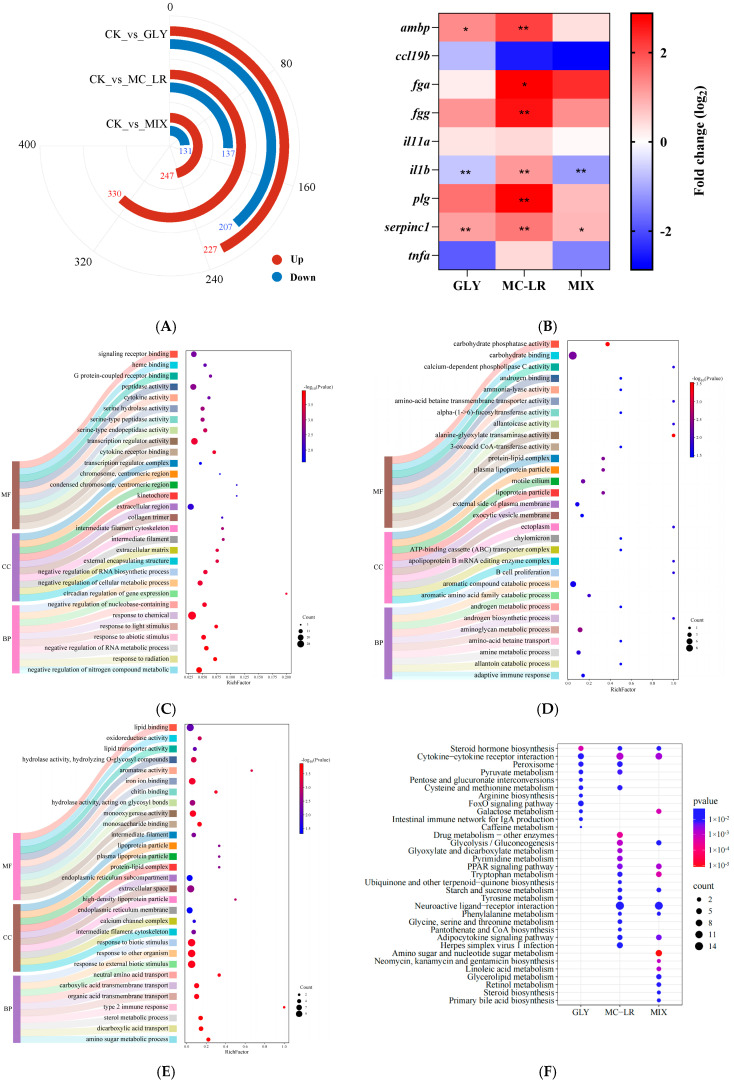

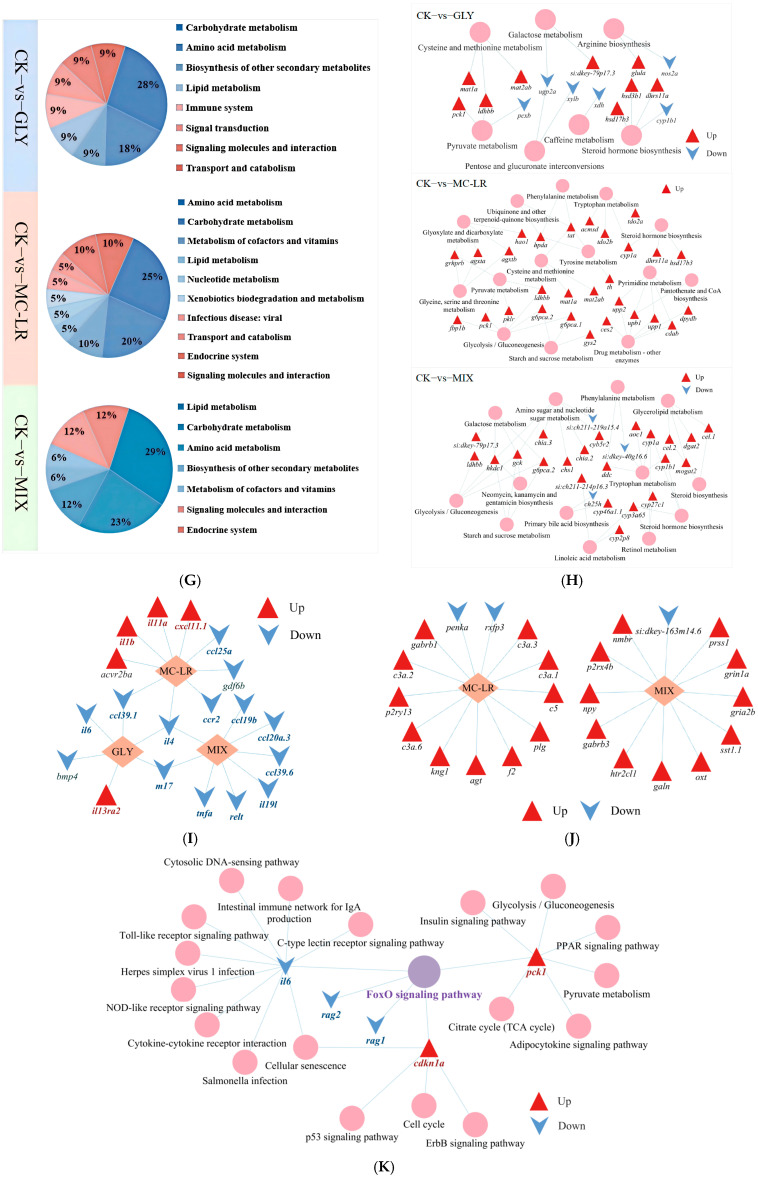
Transcriptional changes in zebrafish gills following GLY and MC-LR exposure. Values are presented as the means ± SD (*n* = 3). Asterisks denote significant differences compared to the controls (* *p* < 0.05, ** *p* < 0.01). (**A**) The number of DEGs in the GLY, MC-LR, and MIX groups. (**B**) Gene expression detection by qPCR. (**C**) TOP 30 GO categories pattern of the DEGs in fish gills after exposure to GLY. (**D**) TOP 30 GO in MC-LR groups. (**E**) TOP 30 GO in MIX groups. (**F**) KEGG analysis of DEGs in the GLY, MC-LR, and MIX groups. (**G**) Classification information of annotated KEGG terms enriched for DEGs. (**H**) The metabolic pathways in the gills of zebrafish following exposure to GLY, MC-LR, and MIX. (**I**) The DEGs in the cytokine–cytokine receptor interaction pathway. (**J**) The neuroactive ligand–receptor interaction pathway analysis in the MC-LR and MIX groups. (**K**) The FoxO signaling pathway analysis in GLY groups. The purple circles represent key signaling pathways, pink circles indicate KEGG pathways, orange diamonds indicate exposure concentration groups, red triangles indicate upregulated genes involved in KEGG pathways, blue arrows indicate downregulated genes involved in KEGG pathways, and grey lines indicate a close relationship between the two.

The sequence data for the gill transcriptome, along with the results of subsequent quality filtering, are summarized in Table S2, Figure S1. A total of 434 (227 increased and 207 decreased), 467 (330 increased and 137 decreased), and 378 (247 increased and 131 decreased) differentially expression genes (DEGs) were identified in the zebrafish gills after GLY, MC-LR, and MIX exposure, respectively (Figure 5A). The expression levels of nine selected DEGs in the gills of fish treated with GLY, MC-LR, and MIX were quantified using qPCR analysis (Figure 5B). Correlation analyses revealed a strong concordance between the qPCR and RNA-seq data for all selected DEGs (Figure S2).

The GO results revealed a total of 327 significantly enrichment terms in the gill of fish following GLY exposure, comprising 208 biological process (BP), 20 cellular component (CC), 99 molecular function (MF), with the top 30 enrichment terms detailed in Figure 5C and Table S3. In the MC-LR group, 277 BP, 18 CC, and 86 MF terms were enriched, with the top 30 terms are summarized in Figure 5D and Table S4. Following MIX exposure, 216 BP, 23 CC, and 108 MF terms were also enriched, and the top 30 terms are detailed in Figure 5E and Table S5.

The KEGG analysis revealed significant enrichment in 11 pathways within the GLY groups (Figure 5F), such as steroid hormone biosynthesis, cytokine–cytokine receptor interaction and peroxisome (Table S6). Additionally, 20 pathways were significantly enriched in the MC-LR groups (Figure 5F), including the PPAR signaling pathway, neuroactive ligand–receptor interaction, and adipocytokine signaling pathway (Table S7). Furthermore, 17 pathways were significantly enriched in the MIX groups (Figure 5F), encompassing amino sugar and nucleotide sugar metabolism, tryptophan metabolism, and galactose metabolism (Table S8).

The steroid hormone biosynthesis pathway and the cytokine–cytokine receptor interaction pathway were significantly enriched in fish gills exposed to GLY, MC-LR, and MIX (Figure 5F), and the DEGs were predominantly upregulated in the steroid hormone biosynthesis pathway (Table S9) and downregulated in the cytokine–cytokine receptor interaction pathway (Figure 5I). In addition to the steroid hormone biosynthesis pathway, 6, 13, and 12 other metabolic pathways were significantly enriched in the gills of zebrafish following exposure to GLY, MC-LR, and their combination, respectively (Figure 5G,H). The neuroactive ligand–receptor interaction pathway was significantly enriched in both the MC-LR and MIX treatment groups (Figure 5J), while the FoxO signaling pathway was only significantly enriched in GLY group (Figure 5K).

## 4. Discussion

Fish gills are frequently regarded as the primary site directly exposed to xenobiotic pollutants. Moreover, gills exhibit high sensitivity and vulnerability to environmental changes, which can compromise their tissue structure and physiological function, potentially impacting fish survival or leading to mortality [32]. The structure of gills is highly sensitive to environmental changes and is commonly utilized as an indicator to assess aquatic environmental pollution [33]. In the present study, exposure to GLY and MC-LR, either individually or in combination, induces epithelial hyperplasia and edema in the gill lamellae, potentially increasing the distance between blood and water, which may serve as a defense mechanism against waterborne pollutions [34]. However, severe epithelial hyperplasia and edema can also lead to inadequate blood oxygen supply, resulting in hypoxia and subsequently accelerating tissue damage [35], as observed in this study. Although the subtle differences in pathological injury among the various exposed groups were not significantly discernible, the present study also demonstrated that exposure to GLY and MC-LR caused damage to gill tissues [36,37]. The results of the SEM analysis clearly revealed distinct alterations in the gill tissue structure across different exposure groups, which may be attributable to the varying toxic mechanisms affecting the gills associated with different pollutants. Although further investigation is required to fully elucidate these mechanisms, the SEM findings, in conjunction with the H&E results, confirm that both GLY and MC-LR, either individually or in combination, are capable of inducing structural damage to zebrafish gills. This gill damage may directly or indirectly impair the normal physiological function of gill tissue, thereby potentially affecting the metabolism and overall health of zebrafish [38].

The Na^+^-K^+^-ATPase, a highly conserved enzyme located in the basolateral membrane of gill epithelial cells, plays an essential role in maintaining ion homeostasis at cellular, organ, and organism levels through its ATP hydrolysis activity [39]. Previous studies have shown that exposure of fish to xenobiotics can reduce the activity of Na^+^-K^+^-ATPase in the gills, potentially inducing cellular stress, compromising the immune system, and causing damage to cells, tissues, and organs [40,41]. In this study, a significant reduction in the activity of Na^+^-K^+^-ATPase was observed in zebrafish gills exposed to GLY, MC-LR, or their combination, indicating that exposure to GLY and MC-LR may impair ion pump function, thereby disrupting normal physiological functions of the gills. Furthermore, the decline in Na^+^-K^+^-ATPase activity is likely attributable to membrane lipoperoxidation damage induced by free radicals [42,43].

Under normal physiological conditions, the level of oxidative stress in organisms is maintained in a dynamic equilibrium. However, exposure to exogenous pollutants may disrupt this redox balance, resulting in free radical-mediated oxidative injury [44]. Previous studies have shown that exposure to either GLY or MC-LR induces excessive production of ROS, leading to oxidative stress even oxidative injury in multiple organs of fish, and oxidative stress is considered one of the key toxic mechanisms through which GLY and MC-LR exert their effects on organisms [45,46,47]. In this study, the results of ROS detection suggest that individual and combined exposures to GLY and MC-LR can disrupt redox balance in the gills, with the combined exposure exerting a more pronounced effect as the duration of exposure increases. The elevated ROS levels may induce oxidative damage by attacking DNA, cellular membranes, and organelle lipids within the gills [48]. 8-OHdG is a critical product resulting from DNA damage under stressful conditions and is widely acknowledged as a sensitive marker for assessing DNA injury [49]. In this study, elevated levels of 8-OHdG were found in the gills following both MC-LR and combined exposures, which is consistent with the phenomenon observed in the brains of MC-LR-treated tadpoles [50]. These findings indicated that the oxidative DNA damage of gills in the combined exposure group may be predominantly attributable to the toxic effects of MC-LR, although further research is needed to elucidate its specific mechanism. MDA, a key end product of lipid peroxidation, functions as a reliable biomarker for assessing oxidative injury [51]. Previous studies have shown that exposure to GLY increases MDA levels in the gills and causes oxidative damage in tilapia [52]. Exposure to MC-LR can induce lipid peroxidation and lead to oxidative damage in the gills of the fish *Brycon amazonicus* [53]. In this study, MDA content in the gills was significantly elevated following exposure to GLY and MC-LR, either individually or in combination, indicating that lipid peroxidation has occurred in zebrafish gills, which may represent a critical pathway contributing to structural and functional impairment of this tissue.

Excessive ROS can promote the production of proinflammatory cytokines, thereby initiating an inflammatory response [54,55]. TNF-α and IL-1β are crucial proinflammatory cytokines and are commonly recognized as key biomarkers of inflammation [56]. In this study, TNF-α levels in the gills were significantly elevated after 21 d of combined exposure to GLY and MC-LR. Furthermore, IL-1β levels exhibited an increasing trend at 7 d and were significantly higher at 21 d across all treatment groups. These results indicate that an inflammatory response occurred in the gills, which may partially explain the structural and functional damage of gills induced by GLY and MC-LR exposure. This is because inflammation serves as a critical component of the host’s innate defense strategy against tissue injury [57]. The innate immunity of fish serves as the primary defense mechanism against external stimuli. Fish have a well-developed complement system, which plays a crucial role in their innate immune response, with C3 being the most abundant component [58]. As a natural antibody, IgM also contributes significantly to the innate immune defense of fish by offering immediate protection against potential environmental threats [59]. In this study, the levels of both C3 and IgM were generally elevated in all three treatment groups. Notably, statistically significant increases were observed in the MC-LR and combined exposure groups at 7 d, but not at 21 d. This suggests that during early stages of exposure, the gills may activate innate immune responses to counteract the threats posed by GLY and MC-LR. However, with prolonged exposure, the effectiveness of these responses appears to decline, although further research is needed to clarify the underlying mechanisms.

To further reveal the specific mechanisms underlying the individual and combined effects of GLY and MC-LR on zebrafish gills, RNA-seq analysis was performed. The results demonstrated that GLY and MC-LR, administered individually or in combination, induced extensive transcriptional changes that may affect various biological functions in zebrafish gills. Moreover, KEGG pathway analysis revealed that the steroid hormone biosynthesis pathway was significantly enriched in the gills of fish exposed to GLY, MC-LR, or their mixture. DEGs in this pathway were predominantly upregulated, suggesting that both individual and combined treatments may promote steroid hormone biosynthesis. Given the critical regulatory role of steroid hormones in osmoregulation, metabolism, and immune responses, these findings imply that GLY and MC-LR exposure could potentially influence these physiological processes in zebrafish gills [60]. In addition to the steroid hormone biosynthesis pathway, 6, 13, and 12 other metabolic pathways were significantly enriched in the gills of zebrafish after exposure to GLY, MC-LR, and their combination, respectively. The majority of these pathways were upregulated, indicating that exposure to these compounds enhanced metabolic activity in the gills, potentially serving as an adaptive response to supply additional energy under stress induced by GLY and MC-LR stress, either separately or in combination.

Meanwhile, the peroxisome signaling pathway was significantly enriched in both the GLY and MC-LR single treatment groups. The PPAR signaling pathway and adipocytokine signaling pathway were notably enriched in both the MC-LR single and GLY and MC-LR combined exposure groups. These pathways play essential roles in regulating lipid metabolism [61], suggesting that exposure to GLY and MC-LR, either individually or in combination, may interfere with normal lipid metabolism in zebrafish gills. Disruption of lipid metabolism is associated with the onset of inflammation and oxidative stress, and in turn, oxidative damage and inflammation may exacerbate lipoperoxidation [62]. Therefore, we speculate that altered lipid metabolism may represent one of the potential mechanisms underlying GLY and MC-LR induced oxidative damage and inflammatory responses in gills.

In the present study, the cytokine–cytokine receptor interaction pathway, a critical signaling involving various immune-related molecules and interactions, was significantly enriched and altered in the gills after exposure to GLY and MC-LR, either individually or in combination. This pathway serves as a critical immune signaling mechanism regulated by a diverse array of cytokines and plays an essential role in both innate and adaptive immune responses [63]. Chemokines, a large family of small cytokines, mediate the directed migration of leukocytes to sites of infection and can also induce the expression of multiple inflammatory cytokines, including ILs and TNFs, thereby enhancing host defense mechanisms [64]. In the present study, although certain chemokines (e.g., *cxcl11.1* in the MC-LR group) and inflammatory cytokines (e.g., *il13ra2* in the GLY group; *il11a* and *il1b* in the MC-LR group) were significantly upregulated, the overall expression levels of chemokines (e.g., *ccl39.1* in the GLY group; *ccl25a*, *ccl39.1*, and *ccr2* in the MC-LR group; *ccl20a.3*, *ccl19b* and *ccl39.6* in the MIX group) and inflammatory cytokines or their receptors (e.g., *il4* and *il6* in the GLY group; *il4* in the MC-LR group; *il19l*, *tnfa*, *m17*, *relt* and *il4* in the MIX group) were significantly reduced. Moreover, the cytokine–cytokine receptor interaction pathway was downregulated across all treatment groups, suggesting that exposure to GLY and MC-LR, either alone or together, may suppress immune responses in zebrafish. Alternatively, the observed decrease might reflect excessive consumption of chemokines and inflammatory cytokines by the gills to counteract exposure-induced stress, potentially representing a protective response in fish. Another significantly enriched signaling pathway, the neuroactive ligand–receptor interaction, was identified in both the MC-LR and MIX treatment groups, with 13 (3 receptors and 10 ligands) and 12 genes (7 receptors and 5 ligands), respectively, showing predominantly upregulated expression. These findings suggest that treatment with MC-LR and MIX enhances signal transduction efficiency in the gills, potentially serving as an adaptive protective mechanism in zebrafish.

In this study, GLY treatment significantly promoted the expression of *cdkn1a* and *pck1* while downregulating *il6*, *rag1*, and *rag2*. These changes in gene expression led to a significant enrichment of the FoxO signaling pathway. The FoxO signaling pathway plays a crucial role in various cellular biological processes, including oxidative stress, inflammation, apoptosis, cell cycle regulation, cell differentiation, proliferation, and metabolic control [65]. *Cdkn1a* acts as a key regulator of the cell cycle [66], and *pck1* is critically involved in carbohydrate and lipid metabolism, as well as other metabolic processes [67]. IL-6 has been shown to promote phagocyte proliferation [68], while *rag1* and *rag2* are essential for V(D)J recombination of immunoglobulin and T-cell receptor genes, which is vital for the adaptive immune response in vertebrates [69]. Therefore, it is speculated that the aforementioned genes alterations within the FoxO signaling pathway contribute to GLY-induced gill injury by modulating metabolic processes, disrupting the cell cycle regulation, and impairing innate immunity.

In the combined exposure groups, seven pathways were identified that were also present in the MC-LR treatment groups but absent in the GLY exposure groups, speculating that the alterations in these pathways within the combined exposure groups may primarily result from MC-LR. Furthermore, seven unique metabolic signaling pathways were identified exclusively in the combined exposure groups, which may be attributed to the combined toxic effects of GLY and MC-LR. These findings indicate that cells may integrate signals from each chemical and generate a novel response profile rather than merely exhibiting a simple additive effect, highlighting the critical importance of evaluating the combined toxicity of environmental pollutants.

## 5. Conclusions

**Figure 6:**
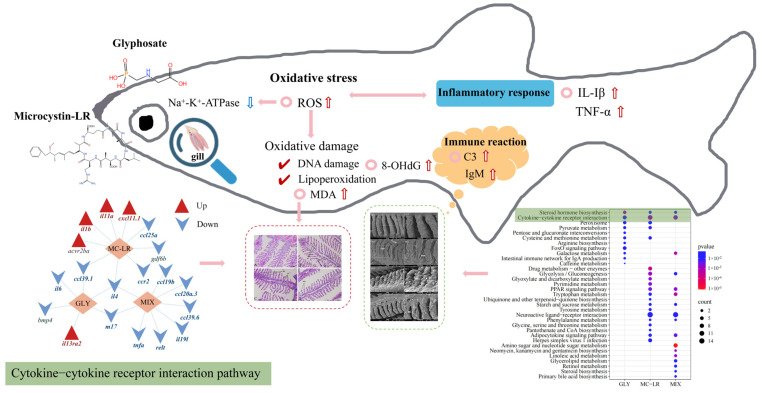
A summary of gill toxicity on zebrafish induced by GLY and MC-LR. The summary figure presents the structural damage to the fish gills caused by GLY, MC-LR alone, and their combined exposure (H&E staining, SEM), as well as the changes in oxidative stress (ROS), inflammation (IL-1β, TNF-α), and immune-related factors (C3, IgM). Additionally, the KEGG pathways are included, and the steroid hormone biosynthesis pathway and the cytokine–cytokine receptor interaction pathway are marked and explained. The red arrow indicates upregulated; the blue arrow indicates downregulated.

In summary, our findings demonstrate that both individual and combined exposures to GLY and MC-LR induce toxic effects on gill structure, which may significantly impair their physiological functions and ultimately threaten fish survival. Oxidative stress, inflammatory responses, and lipid metabolism disruption are likely contributors to these effects. Furthermore, RNA-seq results support the involvement of complex molecular mechanisms underlying the toxic impacts of GLY and MC-LR, both individually and in combination exposure, as illustrated in Figure 6. These findings underscore that the combined toxicity of GLY and MC-LR may involve cells integrating information from each chemical to generate a novel response profile, rather than merely exhibiting an additive effect, underscoring the importance of assessing the combined toxicity of pollutants in aquaculture water bodies. Future studies should aim to clarify the specific biological roles and interactions of key molecular events, such as lipid metabolism disruption and cytokine signaling pathways, in the combined toxic effects of these compounds. Such research will provide a robust theoretical basis for understanding their toxicological mechanisms and informing effective health protection strategies.

## Data Availability

Data supporting the reported results are contained within the article.

## Supplementary Materials

The following supporting information can be downloaded at https://www.mdpi.com/article/10.3390/ani15162355/s1: Methods S1: RNA-seq analysis; Methods S2: Quantitative PCR (qPCR); References [70,71,72,73] are cited in the supplementary materials. Table S1: Nucleotide sequences of qPCR primers used in the present study; Table S2: Summary of sequence data generated from the transcriptome and mass spectrometry analysis of zebrafish gills exposed to GLY, MC-LR, and MIX; Table S3: The top 30 GO analysis on DEGs of gills after zebrafish exposure to GLY; Table S4: The top 30 GO analysis on DEGs of gills after zebrafish exposure to MC-LR; Table S5: The top 30 GO analysis on DEGs of gills after zebrafish exposure to MIX group; Table S6: Significantly enriched KEGG pathways of DEGs in zebrafish gills after exposure to GLY; Table S7: Significantly enriched KEGG pathways of DEGs in zebrafish gills after exposure to MC-LR; Table S8: Significantly enriched KEGG pathways of DEGs in zebrafish gills after exposure to MIX; Table S9: Expression of DEGs in steroid hormone biosynthesis pathway; Figure S1: Transcriptional changes in zebrafish gills following exposure to GLY and MC-LR; Figure S2: Transcriptional changes in zebrafish gills following exposure to GLY and MC-LR. (A) Gene expression of the RNA-seq results. (B) Correlation analysis between qPCR and RNA-seq data in GLY groups. (C) MC-LR groups. (D) MIX groups.
