# Supplementary material for: Adverse Effects of Glyphosate and Microcystin-LR on Fish Health: Evidence from Structural and Functional Impairments in Zebrafish Gills
Source: Animals (Basel). 2025 Aug 11;15(16):2355. doi: 10.3390/ani15162355 (PMC12383198; doi:10.3390/ani15162355)
Supplement: Supplementary file 1 [file animals-15-02355-s001.zip › animals-3745729-supplementary.pdf]

Supplementary Materials

Adverse effects of glyphosate and microcystin-LR on fish health: Evidence from structural and functional impairments in zebrafish gills

Yidan Zhang [1,†], Han Hu [1,†], Linmei Song [1], Zhihui Liu [1], Junguo Ma[1,2,*], and Xiaoyu Li [2]

[1] Pingyuan Laboratory, State Key Laboratory of Antiviral Drugs, College of Life Science, Henan Normal University, Xinxiang, Henan 453007, China; zyd123456202202@163.com (Y. Z.); huhanhh666@163.com (H. H.); 18238796595@163.com (L. S.); liuzh2958@163.com (Z. L.)

[2] Henan International Joint Laboratory of Aquatic Toxicology and Health Protection, Henan Normal University, Xinxiang, Henan 453007, China; 041035@htu.edu.cn (X. L.)

* Correspondence: majunguo@htu.edu.cn (J. M.)

† These authors contributed equally to this work.

1. Supplementary Methods

Methods 1. RNA-seq analysis

Total RNA was extracted from the zebrafish gills using Trizol Reagent (Invitrogen Life Technologies). The concentration, purity, and integrity of the RNA were assessed using a NanoDrop spectrophotometer (Thermo Scientific). RNA sequencing libraries were constructed using the NEBNext Ultra II RNA Library Prep Kit (Illumina) according to the manufacturer's instructions. Transcriptome sequencing and subsequent bioinformatics analysis were conducted by Shanghai Personal Biotechnology Co., Ltd.

DEGs were identified using DESeq (v1.38.3) [70], with a significance threshold of adjusted p value < 0.05 and |log2 (FoldChange)| > 1. Hierarchical clustering was performed to visualize the expression patterns of DEGs. Additionally, Gene Ontology (GO) enrichment analysis and Kyoto Encyclopedia of Genes and Genomes (KEGG) pathway enrichment analysis were conducted using R, based on the hypergeometric distribution [71].

Methods 2. Quantitative PCR (qPCR)

The gill tissues of zebrafish were collected for RNA extraction using the TRNpure Reagent Plus (#NR201-100, Bioconnet, Beijing, China). The quality and concentration of the total RNA were assessed using a NanoDrop spectrophotometer (N50, Implen, Munich, Germany). Subsequently, the HiFiScript cDNA Synthesis Kit (#CW2569M, Cwbio Bio., Beijing, China) was employed to reverse transcribe the RNA into cDNA. Quantitative PCR (qPCR) was performed using a 2×SYBR Premix WizTaq II kit (#NQ811, Nobelab Biotech. Co., Ltd., Beijing, China) to confirm the expression of target genes. The qPCR primers are listed in Table S1. All qPCR experiments strictly followed the Minimum Information for Publication of Quantitative Real-Time PCR Experiments (MIQE) guidelines [72]. The gapdh gene was selected as the reference gene, and the relative expression levels of the target genes in zebrafish gills were quantified using the $2^{-\triangle\triangle Ct}$ method [73].

2. Supplementary Tables

Table S1. Nucleotide sequences of qPCR primers used in the present study.

Primers	Sequences	References
ambp	Forward: 5′- GGCAACCAGAACAACTTCCC -3′<br>Reverse: 5′- AGGACGAATCAAAGGCCCAC -3′	NM_201118.2
ccl19b	Forward: 5′- CGGCGAATCCCTCAGAAAGT -3′<br>Reverse: 5′- ACCCAGTTATTCTGGCTCGG -3′	NM_001302701.1
fga	Forward: 5′- CTACGCACATGCAAAGGCTC -3′<br>Reverse: 5′- GCATGGCGTTCAGATTGTCC -3′	NM_001194989.1
fgg	Forward: 5′- ACGACTTTGGTGACGATCCC -3′<br>Reverse: 5′- CGGTTCATCCACCAACCAGA -3′	NM_213054.1
il11a	Forward: 5′- CCAGCACCATTTTGAGTGGC -3′<br>Reverse: 5′- AGAGTTTACGAGTCCCCCGA -3′	XM_693882.9
il1b	Forward: 5′- CGGGCAATATGAAGTCACC -3′<br>Reverse: 5′- GTCCACATCTCCAGCCTGA -3′	NM_212844.2a
plg	Forward: 5′- GGACTCTCAGCAACCCCAAA -3′<br>Reverse: 5′- GATGGACTGGTGGTGAAGCA -3′	NM_201472.2
serpinc1	Forward: 5′- CCATTGAAGGTGCAACCCTG -3′<br>Reverse: 5′- TCCTCAACACGGAAACGAGG -3′	NM_182863.1
tnfa	Forward: 5′- TACGGAGGCAAAAAGCCACT -3′<br>Reverse: 5′- GTCTGTGCCCAGTCTGTCTC -3′	NM_212859.2
gapdh	Forward: 5′- GAGGGACCCAGCCAACATTA -3′<br>Reverse: 5′- GACTCTCTTTGCACCACCCT -3′	NM_001115114.1

Table S2. Summary of sequence data generated from the transcriptome and mass spectrometry analysis of zebrafish gills exposed to GLY, MC-LR, and MIX.

Sample	Raw Reads	Raw Bases	Clean Reads	Clean Data	Clean Data	Q30	GC
CK1	40.75M	6.15G	40.31M	6.07G	98.72%	96.54%	43.10%
CK2	40.97M	6.19G	40.56M	6.11G	98.77%	96.64%	43.17%
CK3	45.13M	6.81G	44.68M	6.73G	98.81%	96.58%	43.10%
GLY1	41.48M	6.26G	41.06M	6.18G	98.73%	96.63%	43.22%
GLY2	42.51M	6.42G	42.03M	6.33G	98.64%	96.44%	43.13%
GLY3	38.65M	5.84G	38.25M	5.76G	98.72%	96.58%	43.22%
MC-LR1	38.96M	5.88G	38.57M	5.81G	98.79%	96.59%	43.31%
MC-LR2	41.15M	6.21G	40.73M	6.13G	98.72%	96.54%	43.18%
MC-LR3	42.29M	6.39G	41.88M	6.31G	98.80%	96.74%	43.05%
MIX1	43.81M	6.61G	43.41M	6.53G	98.77%	96.78%	43.26%
MIX2	47.29M	7.14G	46.83M	7.05G	98.76%	96.65%	43.25%
MIX3	47.12M	7.12G	46.65M	7.03G	98.75%	96.54%	43.10%

Table S3. The top 30 GO analysis on DEGs of gills after zebrafish exposure to GLY.

Term	Category	ID	*p* value
Negative regulation of nitrogen compound metabolic process	Biological process	GO:0051172	1.10×10^{-4}
Response to radiation		GO:0009314	1.21×10^{-4}
Negative regulation of RNA metabolic process		GO:0051253	1.49×10^{-4}
Response to abiotic stimulus		GO:0009628	2.16×10^{-4}
Response to light stimulus		GO:0009416	2.17×10^{-4}
Rresponse to chemical		GO:0042221	2.52×10^{-4}
Negative regulation of nucleobase-containing compound metabolic process		GO:0045934	2.67×10^{-4}
Circadian regulation of gene expression		GO:0032922	3.12×10^{-4}
Negative regulation of cellular metabolic process		GO:0031324	3.38×10^{-4}
Negative regulation of RNA biosynthetic process		GO:1902679	3.53×10^{-4}
External encapsulating structure	Cellular component	GO:0030312	4.10×10^{-4}
Extracellular matrix		GO:0031012	4.10×10^{-4}
Intermediate filament		GO:0005882	1.09×10^{-3}
Intermediate filament cytoskeleton		GO:0045111	1.17×10^{-3}
Collagen trimer		GO:0005581	8.05×10^{-3}
Extracellular region		GO:0005576	9.40×10^{-3}
Kinetochore		GO:0000776	1.03×10^{-2}
Condensed chromosome, centromeric region		GO:0000779	1.03×10^{-2}
Chromosome, centromeric region		GO:0000775	2.43×10^{-2}
Transcription regulator complex		GO:0005667	2.31×10^{-2}
Cytokine receptor binding	Molecular function	GO:0005126	3.11×10^{-4}
Transcription regulator activity		GO:0140110	3.32×10^{-4}
Serine-type endopeptidase activity		GO:0004252	6.46×10^{-4}
Serine-type peptidase activity		GO:0008236	1.35×10^{-3}
Serine hydrolase activity		GO:0017171	1.35×10^{-3}
Cytokine activity		GO:0005125	3.24×10^{-3}
Peptidase activity		GO:0008233	1.99×10^{-3}
G protein-coupled receptor binding		GO:0001664	2.52×10^{-3}
Heme binding		GO:0020037	3.57×10^{-3}
Signaling receptor binding		GO:0005102	2.89×10^{-3}

Table S4. The top 30 GO analysis on DEGs of gills after zebrafish exposure to MC-LR .

Term	Category	ID	*p* value
Adaptive immune response	Biological process	GO:0002250	5.29×10^{-2}
Allantoin catabolic process		GO:0000256	3.52×10^{-2}
Amine metabolic process		GO:0009308	1.58×10^{-2}
Amino-acid betaine transport		GO:0015838	3.52×10^{-2}
Aminoglycan metabolic process		GO:0006022	3.32×10^{-2}
Androgen biosynthetic process		GO:0006702	1.78×10^{-2}
Androgen metabolic process		GO:0008209	3.52×10^{-2}
Aromatic amino acid family catabolic process		GO:0009074	1.29×10^{-2}
Aromatic compound catabolic process		GO:0019439	2.26×10^{-2}
B cell proliferation		GO:0042100	1.78×10^{-2}
Apolipoprotein B mRNA editing enzyme complex	Cellular component	GO:0030895	1.78×10^{-2}
ATP-binding cassette (ABC) transporter complex		GO:0043190	3.52×10^{-2}
Chylomicron		GO:0042627	3.52×10^{-2}
Ectoplasm		GO:0043265	1.78×10^{-2}
Exocytic vesicle membrane		GO:0099501	2.83×10^{-2}
External side of plasma membrane		GO:0009897	2.21×10^{-2}
Lipoprotein particle		GO:1990777	4.50×10^{-3}
Motile cilium		GO:0031514	5.82×10^{-3}
Plasma lipoprotein particle		GO:0034358	4.50×10^{-3}
Protein-lipid complex		GO:0032994	4.50×10^{-3}
3-oxoacid CoA-transferase activity	Molecular function	GO:0008260	3.52×10^{-2}
Alanine-glyoxylate transaminase activity		GO:0008453	3.14×10^{-4}
Allantoicase activity		GO:0004037	1.78×10^{-2}
Alpha-(1->6)-fucosyltransferase activity		GO:0046921	3.52×10^{-2}
Amino-acid betaine transmembrane transporter activity		GO:0015199	1.78×10^{-2}
Ammonia-lyase activity		GO:0016841	3.52×10^{-2}
Androgen binding		GO:0005497	3.52×10^{-2}
Calcium-dependent phospholipase C activity		GO:0050429	1.78×10^{-2}
Carbohydrate binding		GO:0030246	4.56×10^{-3}
Carbohydrate phosphatase activity		GO:0019203	2.91×10^{-4}

Table S5. The top 30 GO analysis on DEGs of gills after zebrafish exposure to MIX group.

Term	Category	ID	*p* value
Amino sugar metabolic process	Biological process	GO:0006040	1.35×10^{-4}
Dicarboxylic acid transport		GO:0006835	1.51×10^{-4}
Sterol metabolic process		GO:0016125	1.75×10^{-4}
Type 2 immune response		GO:0042092	2.30×10^{-4}
Organic acid transmembrane transport		GO:1903825	2.68×10^{-4}
Carboxylic acid transmembrane transport		GO:1905039	2.68×10^{-4}
Neutral amino acid transport		GO:0015804	2.72×10^{-4}
Response to external biotic stimulus		GO:0043207	3.00×10^{-4}
Response to other organism		GO:0051707	3.00×10^{-4}
Response to biotic stimulus		GO:0009607	3.24×10^{-4}
Intermediate filament cytoskeleton	Cellular component	GO:0045111	4.23×10^{-3}
Calcium channel complex		GO:0034704	2.12×10^{-2}
Endoplasmic reticulum membrane		GO:0005789	4.91×10^{-2}
High-density lipoprotein particle		GO:0034364	1.35×10^{-3}
Extracellular space		GO:0005615	2.68×10^{-3}
Endoplasmic reticulum subcompartment		GO:0098827	4.91×10^{-2}
Protein-lipid complex		GO:0032994	3.31×10^{-3}
Plasma lipoprotein particle		GO:0034358	3.31×10^{-3}
Lipoprotein particle		GO:1990777	3.31×10^{-3}
Intermediate filament		GO:0005882	3.97×10^{-3}
Monosaccharide binding	Molecular function	GO:0048029	2.60×10^{-4}
Monooxygenase activity		GO:0004497	3.11×10^{-4}
Hydrolase activity, acting on glycosyl bonds		GO:0016798	2.18×10^{-3}
Chitin binding		GO:0008061	3.84×10^{-4}
Iron ion binding		GO:0005506	3.89×10^{-4}
Aromatase activity		GO:0070330	6.82×10^{-4}
Hydrolase activity, hydrolyzing O-glycosyl Compounds		GO:0004553	6.96×10^{-4}
Lipid transporter activity		GO:0005319	6.00×10^{-3}
Oxidoreductase activity		GO:0016491	8.22×10^{-3}
Lipid binding		GO:0008289	6.71×10^{-3}

Table S6. Significantly enriched KEGG pathways of DEGs in zebrafish gills after exposure to GLY.

KEGG pathway	Second-level KEGG pathway	The top level KEGG pathway	Pathway ID	Corrected *p* value
Galactose metabolism	Carbohydrate metabolism	Metabolism	dre00052	4.32×10^{-2}
Pentose and glucuronate interconversions			dre00040	1.84×10^{-2}
Pyruvate metabolism			dre00620	1.58×10^{-2}
Arginine biosynthesis	Amino acid metabolism		dre00220	2.86×10^{-2}
Cysteine and methionine metabolism			dre00270	1.86×10^{-2}
Caffeine metabolism	Biosynthesis of other secondary [illegible]		dre00232	4.37×10^{-2}
Steroid hormone biosynthesis	Lipid metabolism		dre00140	4.98×10^{-4}
Cytokine-cytokine receptor interaction	Signaling molecules and interaction	Environmental Information Processing	dre04060	1.22×10^{-2}
FoxO signaling pathway	Signal transduction		dre04068	2.94×10^{-2}
Intestinal immune network for IgA production	Immune system	Organismal Systems	dre04672	4.32×10^{-2}
Peroxisome	Transport and catabolism	Cellular Processes	dre04146	1.43×10^{-2}

Table S7. Significantly enriched KEGG pathways of DEGs in zebrafish gills after exposure to MC-LR.

KEGG pathway	Second-level KEGG pathway	The top level KEGG pathway	Pathway ID	Corrected *p* value
Tryptophan metabolism	Amino acid metabolism	Metabolism	dre00380	7.21×10^{-3}
Cysteine and methionine metabolism			dre00270	7.74×10^{-3}
Tyrosine metabolism			dre00350	1.40×10^{-2}
Phenylalanine metabolism			dre00360	2.48×10^{-2}
Glycine, serine and threonine metabolism			dre00260	2.67×10^{-2}
Glycolysis / Gluconeogenesis	Carbohydrate metabolism		dre00010	1.23×10^{-3}
Glyoxylate and dicarboxylate metabolism			dre00630	1.96×10^{-3}
Pyruvate metabolism			dre00620	6.24×10^{-3}
Starch and sucrose metabolism			dre00500	1.29×10^{-2}
Ubiquinone and other terpenoid-quinone biosynthesis	Metabolism of cofactors and vitamins		dre00130	9.86×10^{-3}
Pantothenate and CoA biosynthesis			dre00770	3.42×10^{-2}
Steroid hormone biosynthesis	Lipid metabolism		dre00140	1.52×10^{-2}
Pyrimidine metabolism	Nucleotide metabolism		dre00240	2.58×10^{-3}
Drug metabolism - other enzymes	Xenobiotics biodegradation and metabolism		dre00983	2.72×10^{-4}

PPAR signaling pathway	Endocrine system	Organismal Systems	dre03320	3.39×10^{-3}
Adipocytokine signaling pathway			dre04920	3.64×10^{-2}
Cytokine-cytokine receptor interaction	Signaling molecules and interaction	Environmental Information Processing	dre04060	1.38×10^{-3}
Neuroactive ligand-receptor interaction			dre04080	2.17×10^{-2}
Peroxisome	Transport and catabolism	Cellular Processes	dre04146	9.91×10^{-3}
Herpes simplex virus 1 infection	Infectious disease: viral	Human Diseases	dre05168	4.76×10^{-2}

Table S8. Significantly enriched KEGG pathways of DEGs in zebrafish gills after exposure to MIX.

KEGG pathway	Second-level KEGG pathway	The top level KEGG pathway	Pathway ID	Corrected *p* value
Linoleic acid metabolism	Lipid metabolism	Metabolism	dre00591	1.32×10^{-3}
Glycerolipid metabolism			dre00561	9.30×10^{-3}
Steroid hormone biosynthesis			dre00140	9.88×10^{-3}
Steroid biosynthesis			dre00100	2.81×10^{-2}
Primary bile acid biosynthesis			dre00120	3.08×10^{-2}
Amino sugar and nucleotide sugar metabolism	Carbohydrate metabolism		dre00520	8.25×10^{-6}
Galactose metabolism			dre00052	5.94×10^{-4}
Starch and sucrose metabolism			dre00500	8.34×10^{-3}
Glycolysis / Gluconeogenesis			dre00010	1.92×10^{-2}
Tryptophan metabolism	Amino acid metabolism		dre00380	4.69×10^{-4}
Phenylalanine metabolism			dre00360	1.83×10^{-2}
Neomycin, kanamycin and gentamicin biosynthesis	Biosynthesis of other secondary metabolites		dre00524	1.02×10^{-3}
Retinol metabolism	Metabolism of cofactors and vitamins		dre00830	1.25×10^{-2}
PPAR signaling pathway	Endocrine system	Organismal Systems	dre03320	1.68×10^{-3}
Adipocytokine signaling pathway			dre04920	1.13×10^{-3}
Cytokine-cytokine receptor interaction	Signaling molecules and interaction	Environmental Information Processing	dre04060	1.94×10^{-3}
Neuroactive ligand-receptor interaction			dre04080	1.50×10^{-2}

Table S9. Expression of DEGs in Steroid hormone biosynthesis pathway.

Group	Gene Name	BaseMean	log_2Fold change	*p* value	Regulation
CK_vs_GLY	*dhrs11a*	17.2362	1.8286	2.61×10^{-3}	Up regulation
	hsd17b3	36.5277	1.1995	2.65×10^{-3}	Up regulation
	cyp1b1	670.807	-1.406	4.59×10^{-3}	Down regulation
	hsd3b1	15.8155	1.3978	4.82×10^{-2}	Up regulation
CK_vs_MC_LR	*dhrs11a*	14.9792	1.5409	2.65×10^{-2}	Up regulation
	cyp1a	11628.5392	1.4965	4.41×10^{-3}	Up regulation
	hsd17b3	39.0915	1.3116	3.46×10^{-4}	Up regulation
CK_vs_MIX	*cyp1b1*	1799.241	1.2567	2.21×10^{-7}	Up regulation
	cyp3a65	108.0445	1.3284	9.42×10^{-3}	Up regulation
	cyp1a	11618.2563	1.35	1.68×10^{-2}	Up regulation

3. Supplementary Figures

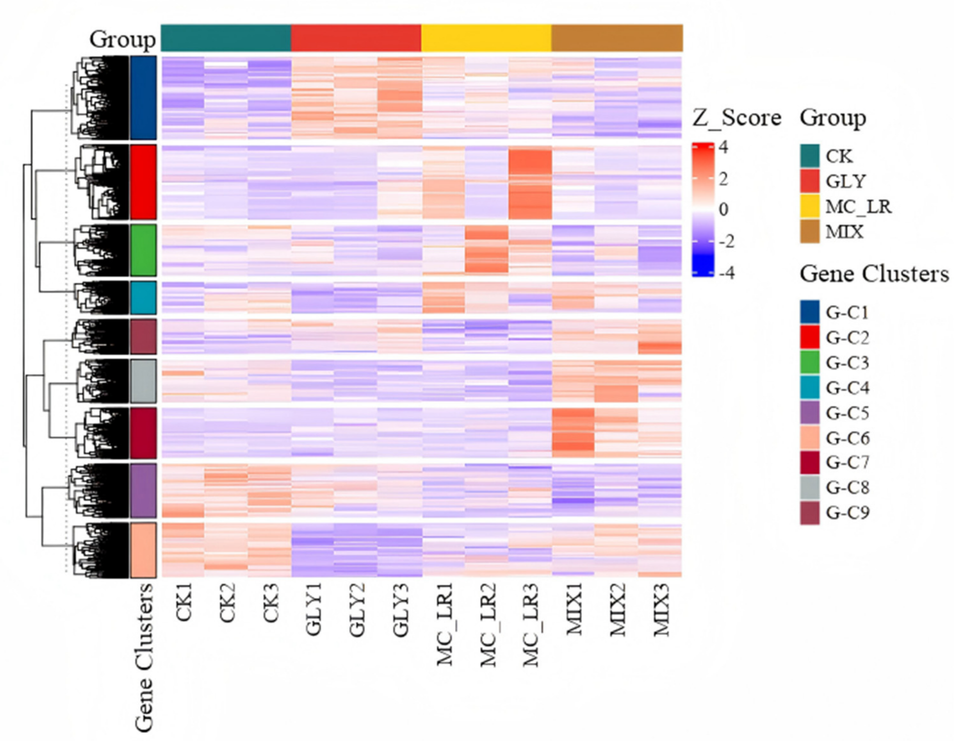

Figure S1. Clustering analysis in the transcriptome in zebrafish gills following exposure to GLY and MC-LR.

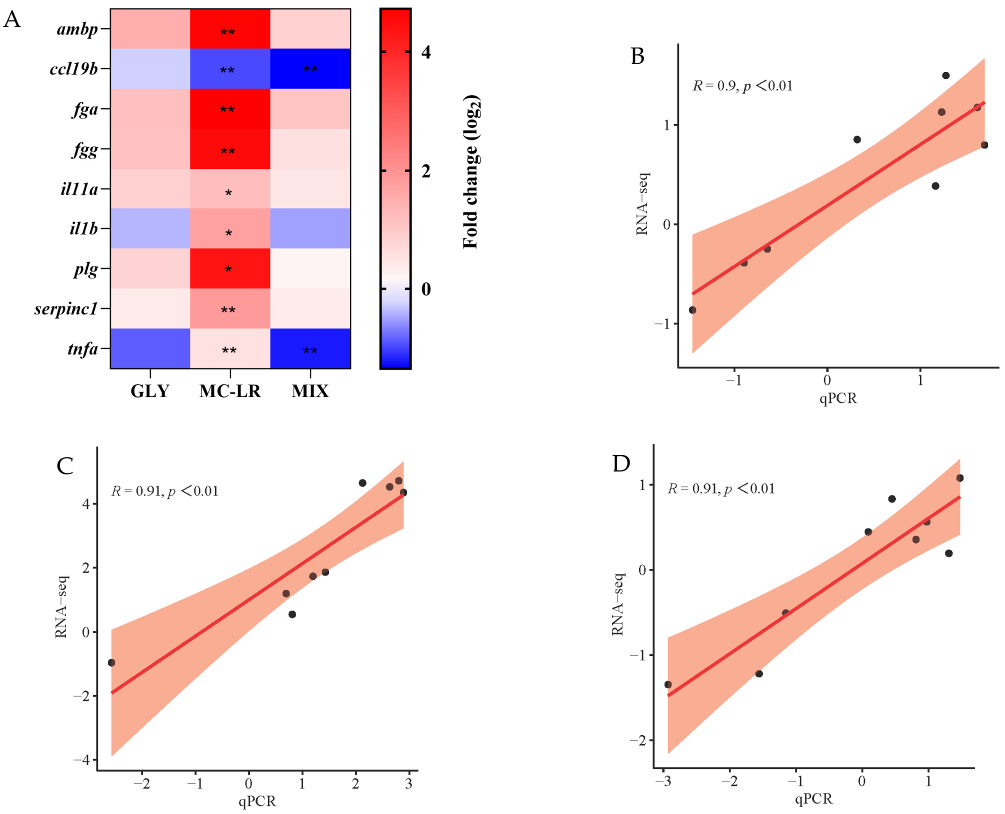

Figure S2. Transcriptional changes in zebrafish gills following exposure to GLY and MC-LR. (A) Gene expression of the RNA-seq results. (B) Correlation analysis between qPCR and RNA-seq data in GLY groups. (C) MC-LR groups. (D) MIX groups.
